# Telemedicine in Resource-Limited Settings to Optimize Care for Multidrug-Resistant Tuberculosis

**DOI:** 10.3389/fpubh.2019.00222

**Published:** 2019-08-13

**Authors:** G. Khai Lin Huang, Gibson Pawape, Magdalene Taune, Stenard Hiasihri, Pilar Ustero, Daniel P. O'Brien, Philipp du Cros, Steve Graham, Richard Wootton, Suman S. Majumdar

**Affiliations:** ^1^Tuberculosis Elimination and Implementation Science Group, Burnet Institute, Melbourne, VIC, Australia; ^2^Daru General Hospital, National Department of Health, Daru, Papua New Guinea; ^3^Department of Infectious Diseases, Barwon Health, Geelong, VIC, Australia; ^4^Norwegian Centre for Integrated Care and Telemedicine, University Hospital of North Norway, Tromsø, Norway; ^5^Faculty of Health Sciences, University of Tromsø, Tromsø, Norway

**Keywords:** tuberculosis, telemedicine, multidrug-resistant, resource-limited, clinical expert group, consilium, digital health

## Abstract

The emergence and spread of multidrug-resistant tuberculosis (MDR-TB) poses a major threat to the global targets for TB control. In recent years, an evolving science and evidence base for MDR-TB has led to much needed changes in international guidelines promoting the use of newer TB drugs and regimens for MDR-TB, however, there remains a significant implementation gap. Due to the complexity of treating MDR-TB, management of cases is often supported by an expert multidisciplinary team, or clinical expert group. This service is often centralized, and may be delivered through a telemedicine platform. We have implemented a Web-based “store-and-forward” telemedicine service to optimize MDR-TB patient care in Daru, a remote and resource limited setting in Papua New Guinea (PNG). From April 2016 to February 2019, 237 cases were discussed using the service. This encompassed diagnostic (presumptive) and treatment cases, and more recently, support to the scale up of preventative therapy for latent TB infection. There were 75 cases in which the use of Bedaquiline was discussed or mentioned, with a high frequency of discussions occurring in the initial period (26 cases in the first 12 months), which has appeared to decrease as clinicians gained familiarity with use of the drug (15 cases in the last 12 months). This service has supported high quality clinical care and fostered collaboration between clinicians and technical experts in a shared learning environment.

## Introduction

Tuberculosis (TB) is one of the top 10 causes of death globally and is the leading cause of death from a single infectious agent. It is a disease that disproportionally affects low- and middle- income countries, where it remains a major public health issue (1). The ambitious goal of the World Health organization's (WHO) End TB Strategy (2015–2035) is to eliminate TB as a public health threat, targeting an incidence rate below 10 cases per 100,000 population per year. The three pillars of the strategy are: integrated patient-centered TB care and prevention, bold policies and supportive systems, and intensified research and innovation (2).

The emergence and spread of multidrug-resistant tuberculosis (MDR-TB, defined as resistance to the most effective first-line anti-TB medications: rifampicin and isoniazid) poses a risk to achieving the End TB goals. MDR-TB is a major contributor to mortality and the financial burden of the antimicrobial-resistance threat (3). MDR-TB care is complex and challenging and the burden is highest in settings where health systems have significant challenges. Globally, patient outcomes remain poor, with 55% of those enrolled completing treatment successfully in 2016 (1), although success rates can be much higher in well-resourced settings (4), and optimized programs (1). The treatment duration for MDR-TB is lengthy (9 months to 2 years), with a regimen of potentially toxic medications, making it more complex and more costly to deliver than drug-susceptible TB (1). The WHO recommends a community-based model of care for MDR-TB, which facilitates decentralization and scale-up in high-burden and low resource settings.

In recent years, an evolving science and evidence base for MDR-TB has led to much needed changes in international guidelines promoting the use of newer TB drugs and regimens for MDR-TB, with further changes anticipated in coming years (5). However, there remains a major implementation gap, with significant barriers to the knowledge-delivery (“know-do”) pathway in MDR-TB care for a number of reasons (6). First, knowledge and experience with managing complicated cases and using the newer drugs such as bedaquiline and delamanid is often limited to a few sites. These sites are typically centralized specialist referral hospitals where expertise, training, clinical advice and active drug safety monitoring can be provided. Second, health workers may have limited training or capacity in settings where MDR-TB care is needed most and is often provided at decentralized sites.

Technical assistance and expertise, including clinical support, either within a country or from international partners plays an important role in optimizing MDR-TB in high burden/resource limited settings (7). Telemedicine is one such modality of providing clinical and technical assistance and can potentially have a role in closing the “know-do” gap to improve patient outcomes and quality of care.

We describe the global utility and experience with telemedicine for MDR-TB care and our own experience with the implementation of a Web-based telemedicine platform to support MDR-TB care in a remote setting of Papua New Guinea (PNG). This model has facilitated the delivery of technical assistance to clinicians in the field through a store-and-forward text-based platform suitable for the local context.

## Current Role and Global Experience With Telemedicine in MDR-TB Care

**Figure 1:**
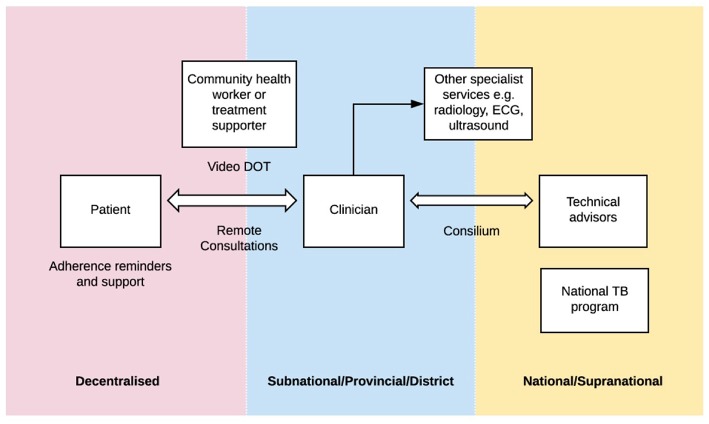
“Digital health in tuberculosis program delivery.” In many countries, TB programs are structured across a number of levels: from the most peripheral (pink) where patients may be located in village or community settings, to subnational levels (blue) where clinicians may be based at provincial or district health centers or hospitals, to the national or supranational level (yellow) where technical advisors and national TB programs are based. There are a number of digital health applications that have the potential to help bridge some of these “gaps”.

Telemedicine is a broad term within the domain of digital health, that encompasses a wide scope of practices, all relating to the delivery of health care at a distance (8). Digital health interventions and innovations have the potential to build upon all three pillars of the End TB Strategy. The WHO has released a digital health strategic agenda (9) describing different approaches. The potential applications of digital health are broad and may include: utilizing innovative approaches such as video-observed therapy to allow for a more patient-centered approach, applications for adherence support, remote patient consultations and remote technical assistance including consensus expert opinions for complex cases (referred to as TB “consilium” in some settings) (see Figure 1).

Due to the complexity of treating MDR-TB, which involves long, complex and expensive regimens and close monitoring for side effects, management of these cases is often supported by an expert multidisciplinary team who provide advice and guidance to the treating clinician. This group has been termed a “TB consilium” in some settings (10), with origins in Eastern European and former Soviet Union TB control programs. TB consilia are typically centralized, with the ability to discuss the most difficult cases at a higher level (e.g., national or supra-national level). In more recent times, as individual members may be remotely located, Web-based telemedicine platforms have been increasingly utilized. This format is typically not “real-time,” but rather a “store-and-forward” arrangement whereby clinicians are able to upload case summaries to seek specific management advice or opinions.

A number of countries, including Belarus, Belgium, France, Mexico, Portugal, and the UK have implemented national level TB consilia (11, 12). These were compared in a recent review (11). Notably, a number of countries were using email-based discussion, which has privacy and legal implications, particularly where identifiable data or clinical images are used (13). Alternately, other countries utilized a regular (monthly) meeting, with attendance either in person or remotely. However, this may not be ideal in terms of timely advice for patient care.

The European Respiratory Society (ERS)/WHO electronic consilium (14) was a supranational, free-to-access, bilingual, Web-based consilium service. Between 2013 and 2018, this system managed more than 400 TB cases from a number of different continents (10). Following the success of the ERS/WHO consilium, a newly promoted initiative: the Global TB Network, was launched in 2018, incorporating a Global TB consilium (15). An additional feature of this network is the provision of support for the management of latent TB infection, an area for which there is increasing focus.

In many medium and high TB incidence settings, TB consilia have acted as an approval or consensus decision system for accessing new drugs or regimens for MDR TB, specifically relating to the use of the new TB drugs: bedaquiline and delamanid (11, 16, 17). Initially, from 2013, where access to these drugs was only via compassionate access or pilot programs, this facilitated a “gatekeeper” function with restricted use for specific indications only. However, with the recent update to WHO treatment guidelines for MDR TB (5), bedaquiline is now recommended upfront for all patients with MDR TB, and hence should no longer require approval for procurement and administration unless supplies are limited and therefore prioritized. In some countries, Delamanid remains on special access mechanisms, such as compassionate use basis and hence consilia remain the main way of approval for accessing the drug.

Finally, consilium can serve an educational role: for clinicians to review how previous similar cases have been managed, and also to have direct access to seek guidance from experienced advisors and mentors. Submitted cases may be rare, unusual or the first experience with a particular regimen or combination of drugs (18), in which case sharing of experience can be beneficial.

### Case Study: Telemedicine to Support MDR-TB Care in Papua New Guinea

Papua New Guinea (PNG) is classified as a high burden country for MDR-TB, TB and TB/HIV by the WHO. It has the highest estimated TB incidence rate in the Western Pacific region at 432 per 100,000 people (1). An unprecedented outbreak of MDR-TB has been reported on Daru Island in the Western Province of PNG (19). This led to the establishment of the emergency response task force for MDR-TB in 2014, led by the National Department of Health in PNG with support from the Australian Government, across three identified “hotspot” provinces, including Daru, Western Province. The programmatic interventions during the response in Daru 2014-17 and clinical care model have been described elsewhere (20, 21).

The Burnet Institute, based in Australia, has been the technical assistance partner in the multi-stakeholder response in Western Province through the RID-TB project (Reducing the Impact of Drug-resistant TB in Western Province) since August 2014. The RID-TB project supports the provincial TB program in program design and evaluation, implementation, clinical care, capacity building and training, health systems strengthening and operational research. The RID-TB project staff include long term advisors (remote/visiting) and field-based TB specialists. Clinical care is provided by medical officers and health workers at Daru General Hospital. A clinical expert group (CEG) (or consilium) was formed to support MDR-TB care and provide advice, primarily on patients to whom the standardized care pathway did not apply e.g., pediatric cases, extra-pulmonary TB, TB-HIV, extensively drug-resistant TB (XDR-TB) and management of adverse effects or complex cases. In particular, there was a need to provide clinical consensus decision for patients initiated on newer TB drugs—bedaquiline and delamanid—which were initially obtained via compassionate access, and for which there was limited in-country experience.

Because of the remote location, the CEG initially communicated de-identified case discussions on email and a file server for records. After assessment of various options, the project implemented a telemedicine platform in April 2016: Collegium Telemedicus (22). The choice of platform was based on the considerations of data security and accountability, cost, bandwidth requirements, ease of use, and the capacity for two-way discussions between clinicians and the CEG. The platform allows users to submit case summaries, and also to attach relevant results, to assist in interpretation e.g., X ray images or electrocardiogram (ECG) records. Our CEG is comprised of a group of technical experts with a broad range of international experience, who are also familiar with the Daru context. Because of time limitations and varying availability, the group has a monthly roster, to ensure that there is always a timely response, although areas of expertise can be drawn on for specific cases.

The objectives of the consilium/CEG: are to provide clinical support in complex case management, with the additional functions of capacity building, quality assurance, and the formation of collaborations between physicians from different locations (e.g., Daru General Hospital, Port Moresby General Hospital and the National TB Program).

From April 2016 to February 2019, 237 cases were discussed using the platform. This encompassed diagnostic (presumptive) and treatment cases. More recently, support to the scale up of preventative therapy for latent TB infection has been included. Across this time period, there have been 44 different “user” accounts (either with the capacity to make referrals, or view only for educational purposes), and a current pool of five technical experts providing technical support and advice across a range of areas. The median response time from referral for cases was relatively prompt at 17.6 h, facilitating timely decision making. The median number of messages exchanged for each case was 10, reflecting the amount of user engagement and discussions occurring.

The program has seen a high turnover of field-based staff, due to the remote and challenging context. As a result, there can be some variation in practices with different clinicians and one key benefit of Collegium Telemedicus that we have noted is for continuity of patient care. With patients often encountering several different clinicians across their “journey,” this allows transparency in the rationale for previous decision making. As a learning tool, the consilium has also served as a useful resource to explore how similar patients may have been approached in the past e.g., MDR-TB infections in pregnancy.

The telemedicine service facilitated the uptake of innovations where technical support was required. The programmatic use of the TB drug Bedaquiline was scaled up in PNG in 2016, procured by the National TB Program, and supported by the global donation program (21). In this context, the consilium proved useful in supporting local clinical decision making in the scale up of this drug where there was little previous in-country experience and a limitation on its supply. There were 75 cases in which the use of Bedaquiline was discussed or mentioned, with a high frequency of discussions occurring in the initial period (26 cases in the first 12 months), which has appeared to decrease as clinicians gained familiarity with use of the drug (15 cases in the last 12 months).

A number of progress/feedback reports were completed by program participants. Of the five reports that were completed across 2016–2018, all respondents felt that the responses they received on Collegium Telemedicus were timely and well-adapted to the local environment. However, in response to whether there would be a benefit to the eventual outcome for the patient, two participants responded “yes,” two responded “perhaps” and one responded “no”. Three of the five respondents felt that there was educational benefit in the process.

Although there were a number of concurrent interventions across this time period, we believe that this service has contributed, in part, to the very good outcomes now seen for MDR-TB patients in Daru (20, 21). Treatment success rates for the 2015 MDR TB enrolment cohort were 81%, with only 4% of patients being lost to follow up and 4% failing treatment (20).

An analysis of the perceived benefits and challenges of the telemedicine platform according to the defined objectives, is provided in Table 1. There have been a number of challenges throughout the implementation process including high field-staff turnover and variable engagement depending on staff preferences. Internet access and connectivity has been a limiting issue at times, despite the low bandwidth requirements. While expansion to telemedicine services that incorporate real-time video linkages is an appealing option, it is currently still not possible for many rural sites in PNG.

**Table 1:** Benefits and challenges of the telemedicine platform.

**Domain**	**Benefits**	**Challenges**
Clinical support in complex case management	Expert input within 24–48 h Continuity of care with one record of all discussions Consensus expert opinion for challenging cases Multi-disciplinary expert advice including infectious diseases, pediatrics, radiology, and other specialists Continuity of clinical advice from experts who know and have worked in the program Consensus opinion for treatment initiation with new drugs, bedaquiline, delamanid, initially under compassionate access	Internet access variable requiring considerable time at field level to load cases and responses Variability in initiation of referrals depending on preference of clinical staff in the field Difficult to link patient notes from patients with more than one treatment episode
Capacity building	Inclusion of recent evidence and guidelines within responses Previous cases available for learning by new staff Two-way process with discussions of cases Training for new staff on use of protocols in difficult cases	High turnover of staff in the field limiting longer term capacity building
Quality assurance of clinical practice	Potential for audits of quality of care and expert advice Support of roll out of active drug safety monitoring	Audits not linked in with national quality assurance systems
Formation of collaborations between physicians from different locations	Strengthened links between Daru team and technical assistance partner	System currently only used in one site within PNG

## Conclusion

Implementation of the WHO End TB strategy will require ongoing innovation and the introduction of new treatments and care models for MDR-TB. The WHO guidelines will have a number of significant and ongoing updates as new evidence is generated, in particular for MDR-TB treatment. These will require technical assistance in resource-limited settings, where training and capacity gaps may exist.

The RID-TB technical assistance project in Daru, a remote and resource limited setting in PNG, implemented a telemedicine platform to optimize MDR-TB patient care. The telemedicine platform supported high quality clinical care and fostered collaboration between clinicians and technical experts in a shared learning environment. In particular, it bridged the knowledge-delivery gap in supporting the scale-up of innovations such as implementation of bedaquiline for MDR-TB and management of complex cases.

Telemedicine is a key intervention to optimize patient care and build local capacity and clinical collaborations in the management of MDR-TB in low resourced settings, particularly in the landscape of new and emerging evidence and evolving standards of care in MDR-TB anticipated in the coming decade.

## Data Availability

The datasets for this manuscript are not publicly available. Requests to access the datasets should be directed to khai.huang@burnet.edu.au.

## Author Contributions

GH, GP, RW, PdC, and SM: concept. GH and RW: acquisition of data. GH, GP, DO'B, PdC, RW, and SM: drafting of manuscript. GH, GP, MT, SH, PU, DO'B, PdC, SG, RW, and SM: critical review and final approval of manuscript.

### Conflict of Interest Statement

The authors declare that the research was conducted in the absence of any commercial or financial relationships that could be construed as a potential conflict of interest.
